# Amniotic Mesenchymal Stromal/Stem Cell–Derived Extracellular Vesicles for Equine Chronic Degenerative Endometritis Treatment

**DOI:** 10.1002/vms3.70685

**Published:** 2026-02-05

**Authors:** Giulia Gaspari, Federico Funghi, Carlo Cantile, Francesco Camillo, Duccio Panzani, Saverio Maltinti, Diana Fanelli, Rebecca Moroni, Fausto Cremonesi, Paola Gagni, Anna Lange‐Consiglio

**Affiliations:** ^1^ Department of Veterinary Medicine and Animal Science (DIVAS) Università degli Studi di Milano Lodi Italy; ^2^ Private Practitioner Grosseto Italy; ^3^ Department of Veterinary Science University of Pisa Pisa Italy; ^4^ Institute of Chemical Sciences and Technologies “Giulio Natta” National Research Council SCITEC‐CNR Milano Italy

**Keywords:** chronic degenerative endometritis, extracellular vesicles, foetal–maternal communication, mare, pregnancy

## Abstract

**Background:**

Equine chronic degenerative endometritis (CDE) is a progressive process characterized by endometrial fibrosis that could be responsible for alterations of uterine environment and foetal–maternal communication.

**Objectives:**

The aim of this study was to try to restore this communication by intrauterine administrations of amniotic cell–derived extracellular vesicles (AMC‐EVs) in a case series.

**Study Design:**

Twelve mares were selected on the basis of their reproductive history of early embryonic loss or abortion and clinical suspicion of CDE subsequently verified with histopathological examination of endometrial biopsies.

**Methods:**

Gynaecological and ultrasound examinations and histopathological examination of endometrial biopsies were performed. Mares were divided into two groups: Seven mares in Group 1 received a single treatment cycle (corresponding to two intrauterine AMC‐EV administrations), whereas five mares of Group 2 received two treatment cycles (corresponding to four intrauterine AMC‐EV administrations). Each administration was of 20 billion AMC‐EVs diluted in 50 mL of sterile saline solution.

**Results:**

Eleven mares were able to establish pregnancy after the treatment with AMC‐EVs without significant difference in pregnancy outcomes between one or two treatment cycles (six out of seven mares of Group 1 and all mares of Group 2 were pregnant), suggesting that one cycle may be sufficient. The histological condition of their endometrium did not show any improvement in Kenney–Doig classification, meaning AMC‐EVs did not exert regenerative activity but probably contributed to re‐establishing a functional paracrine interaction between embryo and maternal tissues.

**Main Limitations:**

This study has the limitation of the small number of animals enrolled and the lack of a control group. However, considering the large number of past artificial insemination attempts for each animal enrolled in this study, each mare could be considered self‐control.

**Conclusions:**

It would seem possible that AMC‐EVs supported and enhanced foetal–maternal communication that was compromised by CDE.

## Graphical abstract

Equine endometrium can develop progressive fibrotic and functional alterations, probably correlated with age and inflammatory insults that alter uterine microenvironment, creating hostile conditions for embryo implantation. Named chronic degenerative endometritis, this condition leads to reduced fertility in older mares. Extracellular vesicles secreted by amniotic mesenchymal stromal/stem cells carry molecules with therapeutic and regenerative properties. Their intrauterine administration in 12 mares, with several previous negative insemination attempts, led to an improvement in fertility, probably driving a significant restoration of maternal–foetal talk.

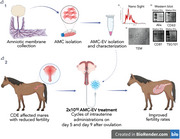

## 1 Introduction

Equine chronic degenerative endometritis (CDE), or endometrosis, is a progressive and severe condition of the endometrium characterized by periglandular fibrosis and alteration of glands and surrounding stroma (Kenney 1978; Hoffmann et al. 2009; Aresu et al. 2012). This irreversible condition may affect single or multiple uterine glands (nests), and their glandular epithelia can remain intact or necrotically degenerate, depending on the nondestructive or destructive nature of the pathology, respectively (Hoffmann et al. 2003; Schöniger and Schoon 2020).

The histological changes typical of CDE include the synthesis of collagen fibres from stromal cells and their differentiation into myofibroblasts, which are responsible for the deposition of extracellular matrix that typically leads to endometrial fibrosis (Hoffmann et al. 2003; Rebordão et al. 2014).

Histopathological examination of endometrial biopsy is currently the best technique to diagnose CDE, as it enables evaluation of endometrial status and integrity. To correlate the histological samples to the alterations of fertility encountered in mares, nowadays a specific classification of the endometrium, developed by Kenney and Doig (1986) and modified by Snider et al. (2011), is widely used. On the basis of the histological appearance and extent of inflammation and fibrosis, the endometrial tissue is classified in different categories correlated to the predicted foaling rate of the mare (Kenney and Doig 1986; Snider et al. 2011; McKinnon et al. 2011; Rebordão et al. 2021).

The pathogenesis and aetiology of CDE are still not fully understood, but mare's age, multiple pregnancies, parturition and repeated or persistent endometrial inflammation might play a consistent role in its development (Ricketts and Alonso 1991). Fibrosis of the stromal tissue, glandular degeneration and a persistent inflammatory condition of the endometrium develop during the ageing process, leading to reduced fertility in older mares (Ricketts and Alonso 1991; Katila and Ferreira‐Dias 2022; Leblanc and Causey 2009; Ebert et al. 2014).

The disfunction of fibrotic glands also leads to both qualitative and quantitative alterations of endometrial secretions (Hoffmann et al. 2009; Hoffmann et al. 2003; Hein 2000), which are essential, in the case of conception, for foetal maintenance and development (Hoffmann et al. 2009). Therefore, an altered uterine microenvironment clearly affects the critical and complex cross‐talk between foetal and maternal tissues, creating hostile conditions for embryo implantation and development. This delicate communication occurs through paracrine signalling and involves several soluble and insoluble factors secreted by cells. Among these mediators, extracellular vesicles (EVs) play an essential role in many stages of conception, ranging from gamete maturation to embryo implantation and survival (Machtinger et al. 2016).

EVs are lipidic bi‐layered particles released by cells in the surrounding environment to exchange information and signals with each other. They transport many functional molecules, including lipids, proteins, DNA and RNAs, which can influence the biological activity of their targeted cells (Camussi et al. 2010; EL Andaloussi et al. 2013; Zaborowski et al. 2015).

Some studies highlighted the active involvement of EVs secreted in oviductal (Al‐Dossary et al. 2013; Harris et al. 2020) and uterine fluid (Ng et al. 2013; Piibor et al. 2023), and in foetal–maternal cross‐talk (Capra and Lange‐Consiglio 2020; Qamar et al. 2020). During the first days of pregnancy, trophectoderm and uterine epithelial cells interact through EV secretion (Burns et al. 2016). Embryos are thought to develop their own microenvironment through the release of paracrine and autocrine factors (Betteridge and Fléchon 1988). It has been demonstrated that Day‐8 equine embryos interact with epithelial oviductal cells actively secreting EVs able to influence their function through the delivery of early pregnancy factor (Hsp10) and microRNAs (miRNAs) (Bemis et al. 2012). From the maternal side, endometrial cells can release miRNAs and adhesion molecules enclosed in EVs, which are delivered to the blastocyst or adjacent uterine cells, influencing endometrial receptivity and implantation (Machtinger et al. 2016). After the implantation phase, placental cells produce EVs able to modulate maternal physiology and foetal development (Redman and Sargent 2008). These EVs are also characterized by immunomodulatory properties (Théry et al. 2009) to avoid foetal rejection by maternal immune system (Asea et al. 2008).

EVs represent a paracrine means of communication also of mesenchymal stromal cells (MSCs) used in regenerative medicine. These cells are a heterogeneous population found in the stroma of various adult, foetal and extra‐foetal tissues where they act as progenitor cells during natural tissue turnover. Extra‐foetal tissues, such as umbilical cord, amniotic fluid, amniotic membrane and placenta, do not have ethical issues related to embryonic cells and do not require invasive procedures such as bone marrow harvesting (Bailo et al. 2004; Lange‐Consiglio et al. 2012). In the last decade, equine amniotic cells (AMCs) were obtained from amniotic membrane, an extra‐foetal tissue which is normally discarded after birth and readily available without the need for invasive procedures (Lange‐Consiglio et al. 2012). In vitro, these cells have the property of modulating the proliferation of peripheral blood mononuclear cells, suggesting their role in immunomodulatory processes (Lange‐Consiglio et al. 2013). They can act both in cell‐to‐cell contact and via paracrine signalling, with the potential either to drive the inflammatory response toward its resolution or to strengthen it, depending on the surrounding microenvironment (Krampera 2011). Indeed, some of our studies have demonstrated that equine AMCs or their derivatives (soluble component of conditioned medium [CM] or EVs) counteract in vitro induced inflammation in macrophages (Zucca et al. 2016), tendon (Perrini et al. 2016) and endometrial cells (Lange‐Consiglio et al. 2016). Other in vivo studies with AMC‐CM in the treatment of spontaneous tendon lesions in horses (Lange‐Consiglio et al. 2013) or mastitis in bovine (Lange‐Consiglio et al. 2019) confirm their therapeutic, regenerative and anti‐microbial properties.

A study by Lange‐Consiglio et al. (2018) identified a series of miRNAs selectively compartmentalized into EVs secreted by AMCs and involved in regulation of inflammatory mechanisms. A recent ulterior study suggested how amniotic cell–derived EVs (AMC‐EVs) may contribute to the prevention of persistent post‐breeding induced endometritis, downregulating pro‐inflammatory cytokines and up‐regulating anti‐inflammatory ones (Lange‐Consiglio et al. 2023).

Assuming that CDE causes a deficit in embryo‐maternal cross‐talk, this case series is an expansion of a previous case report conducted on a Friesian mare affected by category IIB CDE (Lange‐Consiglio et al. 2020) where AMC‐EV intrauterine administration was allowed to improve the histological condition of this mare's uterus and to successfully give birth to a healthy foal. The aim of our study was to confirm, with a larger population, that AMC‐EVs might convey to damaged tissue molecules able to improve endometrial histological classification and pregnancy rates, probably restoring a proper communication between maternal and embryonic counterparts.

## 2 Materials and Methods

The AMC‐EVs were already characterized in a previous work (Lange‐Consiglio et al. 2023) following Minimal Information for Studies of Extracellular Vesicles (MISEV) recommendations (Théry et al. 2018). In this study, EV characterization was carried out by NanoSight, Western blot analysis and transmission electron microscopy (TEM).

### 2.1 Isolation of Amniotic Mesenchymal Cells and Characterization of Their EVs (NanoSight, Western Blot Analysis and TEM)

Three allanto‐amniotic membranes were obtained at the term of a normal and physiological pregnancy and, after fragmentation, were digested with 1 mg/mL collagenase type I and 20 µg/mL DNase (Roche, Mannheim, Germany) for 3 h at 38.5°C.

A pool of isolated amniotic mesenchymal cells (AMCs) was expanded until Passage 3 and then maintained in serum‐free Ultraculture medium (Ultraculture, Lonza, Milan, Italy) in a controlled atmosphere with 90% humidity, 5% CO_2_ and 38.5°C for 3 days. CMs were collected each morning from the culture flasks and replaced with fresh media. CMs were centrifuged at 1600 × *g* for 20 min to discard cells and at 4500 × *g* for 20 min to discard debris and then stored at −80°C until EV isolation.

The obtained CM was ultracentrifuged at 100,000 × *g* (Beckman Coulter OptimaX, Milan, Italy), 4°C for 1 h, and the resulting pellet was resuspended in serum‐free medium. Aliquots of known concentration were stored at −80°C for the subsequent experiments.

The isolated AMC‐EVs were characterized following MISEV guidelines (Théry et al. 2018): NanoSight, dot blot, Western blot analysis and TEM as briefly described.

*NanoSight*: EV size and concentration parameters were obtained using nanoparticle tracking analysis (NTA) performed according to manufacturer's instructions using a NanoSight NS300 system (Malvern Technologies, Malvern, UK) configured with a 532 nm laser.

*Western blot analysis*: EV markers were evaluated using 32 µL of each sample in replicate. The samples were treated with 8 µL of Laemmli buffer in redaction condition, and the preparation was heated for 10 min at 95°C. The samples were run on electrophoretic gel SDS–PAGE (4%–20%, Mini‐Protean TGX Precast protein gel, Bio‐Rad) under an electric field and transferred into the nitrocellulose membrane (BioRad, Trans‐Blot Turbo, Milan, Italy). A blocking step was performed for 1 h with 5% (w/v) BSA in T‐TBS (tris‐buffered saline: 150 mM NaCl, 20 mM Tris–HCl, pH 7.4 and 0.5% Tween 20). After an overnight incubation at 4°C with primary antibodies, membranes were incubated with polyclonal antibody anti‐TSG101 (1:500 dilution, Invitrogen, Monza, Italy), monoclonal antibody anti‐Alix (1:1000 dilution, Santa Cruz, CA, USA), monoclonal antibody anti‐CD81 (1:500 dilution, Santa Cruz, CA, USA) and monoclonal antibody anti‐CD63 (1:500 dilution, BD Biosciences, New Jersey, USA). Strips were washed with TBS‐T, and membranes were incubated with horseradish peroxidase–conjugated anti‐mouse secondary antibody (BioRad) diluted 1:3000 in TBS‐T with 1% BSA. Final washes were performed, and the signal was detected using Bio‐Rad Clarity western ECL substrate (Bio‐Rad) and imaged using a ChemiDoc XRS+ (BioRad).

TEM: A drop of 10 µL EVs with a concentration of 20 × 10^9^ mg/mL was used and was absorbed on 300‐mesh formvar/carbon copper grids. EVs were then fixed with a solution containing 2.5% glutaraldehyde for 5 min. After repeated washing in distilled water, the grids were contrasted with 2% uranyl acetate, air‐dried, and examined using a TEM (Desantis et al. 2019).

### 2.2 Mare Selection and Anamnesis

The study was carried out during the reproductive season of 2024.

Twelve mares (M) enrolled in this case series were selected on the basis of their reproductive history of early embryonic loss or abortion and clinical suspicion of CDE, subsequently verified with endometrial biopsy.

Selected mares ranged in age from 6 to 21 years old. These animals were put into reproductive activity after their retirement from sportive careers and no past reproductive pathologies were reported. M1–M3 were embryo‐receiving mares.

The population of our study includes clinical patients and not experimental or research horses.

Mares’ breeds and medical history are described in Table 1.

**TABLE 1:** Reproductive anamnesis of the mares enrolled.

Mare	Breed	Age (years)	Reproductive history
M1	Trotter	21	Used as embryo‐receiving mare for many years with positive results. Retired from reproduction for age limitations. Negative insemination attempts since 2020
M2	Trotter	20	Last delivery before 2012; retired from reproduction for age limitations. Negative insemination attempts since 2021
M3	Trotter	17	Last delivery in 2015; then retired from reproduction. Negative insemination attempts since 2022
M4	Trotter	10	Last delivery in 2016; negative insemination attempts since 2016
M5	Italian saddle horse	15	Last delivery in 2015; used as embryo‐donor in 2017 with positive results; then negative insemination attempts
M6	Trotter	14	Abortion in 2020 on Month 7; foetal resorption before Day 45 in 2022; then negative insemination attempts
M7	Trotter	13	Last delivery in 2019; embryonic vesicle resorption before Day 25. Negative insemination attempts since 2020
M8	Arabian thoroughbred	12	Last delivery in 2017; then negative insemination attempts during following breeding seasons
M9	Quarter horse	16	Delivery in 2015; negative insemination attempts during the following breeding seasons; pregnancy in 2021 after corticosteroid treatment
M10	Quarter horse	18	No previous deliveries; negative insemination attempts during following breeding seasons
M11	Quarter horse	19	Four previous deliveries; negative insemination attempts since 2018; Caslick intervention for perineal reconstruction after development of vestibular fistula
M12	Quarter horse	6	No previous deliveries; negative insemination attempts during 2020 and 2021 with foetal resorption before Day 45

Considering the large number of past artificial insemination attempts for each mare enrolled in this study, each animal could be considered self‐controlled.

### 2.3 Gynaecological Evaluation

Mares were gynaecologically examined to evaluate the condition of their reproductive tract and to set a standardized experimental treatment according to their oestral phase.

The perineal conformation was visually evaluated to exclude the possible presence of lesions or previous interventions. The integrity of the vestibulo‐vaginal sphincter was also assessed to exclude secondary infections or faecal contaminations.

A 10% iodine povidone solution was used to accurately clean mares’ vulvas. The vaginoscopic examination was performed with a speculum inspection (Kruuse speculum, DLM, Lodi, Italy) that allowed us to evaluate the possible presence of alterations that could cause endometritis, such as lacerations or urine stagnation. Mares’ oestral phase was determined by the characteristics of vaginal vestibulum and cervix.

The oestral phase was further assessed by rectal inspection, evaluating ovaries (shape, dimension, consistency, tenderness), uterus (tone, dimension, consistency) and cervix.

All mares showed good health and nutritional state. Only one mare (M11) presented an altered perineal conformation that caused faecal contamination of vulva and vestibulum. The same mare underwent Caslick intervention in the past (Table 1). All the other mares did not show any functional or anatomical anomality.

### 2.4 Ultrasound Examination

Transrectal ultrasound examination was performed with an Ibex Pro ultrasound machine equipped with a 5 MHz linear probe (VIBI, Mi, Italy) to assess the oestral phase more precisely and to evaluate the presence of intrauterine fluid accumulation.

### 2.5 Endometrial Biopsy

Endometrial biopsies were collected at the beginning of the reproductive season to assess the starting condition of the mares and after the treatments to evaluate possible improvement of uterine histological condition.

Vulvar and perineal areas were accurately washed and disinfected with 10% iodine povidone solution. Sixty cm alligator forceps were used to collect endometrial tissue from the dorsal wall of the uterine body for histological analysis. The sample obtained was immediately transferred into a 10% formalin solution and stored until histological evaluation.

All the endometrial biopsies were dehydrated, included in paraffine, cut into 4 µm sections (Leica RM 2145 microtome, Mi, Italy) to obtain single‐cell layers and then haematoxylin–eosin stained (automated Leica Auto Stainer XL, Mi, Italy) to observe inflammatory infiltrates.

Histological sections were blindly evaluated according to the Kenney–Doig classification, modified by Snider et al. (2011) afterwards.

### 2.6 Therapeutical Protocol

All EVs were derived from a single batch obtained from the same three allanto–amniotic membranes, as previously described. The EVs were cryopreserved, allowing a heterologous use of these products every time it is necessary to carry out cell‐free therapy. All mares were treated with the same batch of AMC‐EVs, eliminating variability in production protocols and variability in initial biological samples (amniotic membranes) that could interfere with clinical outcome.

Twelve mares were randomly divided into two groups: Seven mares (Group 1: M1–M7) received a single‐treatment cycle (corresponding to two intrauterine AMC‐EV administrations), whereas five mares (Group 2: M8–M12) received two treatment cycles (corresponding to a total of four intrauterine AMC‐EV administrations).

*Group 1*: Each intrauterine treatment cycle started on ovulation day (Day 0) that was detected by trans‐rectal ultrasound and clinical examination. On Day 5 after ovulation, mares received the first AMC‐EV intrauterine administration through an infusion of 2 × 10^10^ EVs diluted in 50 mL of sterile saline solution, according to the dosage used in previous studies (Lange‐Consiglio et al. 2023; Lange‐Consiglio et al. 2020). Right before the procedure, vulvo‐perineal area was accurately cleaned and disinfected with 10% iodine povidone solution and subsequently dried. On Day 9, the same procedure was repeated, and during the following days, mares were monitored by ultrasound examination to identify the next ovulation.

Post‐treatment biopsy was performed on Day 10 after the second ovulation post‐treatment, during diestrus.

*Group 2*: These mares started the second treatment cycle of AMC‐EV intrauterine administration on Day 5 after the third ovulation, repeating the same procedure as Group 1.

Group 2 mares received a post‐treatment biopsy on Day 10 after the fifth ovulation following the beginning of the first treatment cycle.

When the first heat appeared (third heat after the last treatment in Group 1), all mares of Groups 1 and 2 were artificially inseminated (AI) with fresh semen by a standard procedure. Different stallions of proven fertility were chosen according to the reproductive programming of each mare. The insemination dose was set at 500 × 10^6^ total spermatozoa, diluted in 30 mL of INRA96.

Mares were examined for conception diagnosis 14 days after ovulation and monitored by ultrasound for the progression of pregnancy during the following months.

**FIGURE 1:**
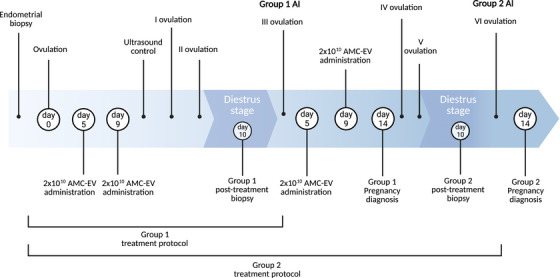
Schematic representation of the therapeutical protocol followed. AI, artificially inseminated; AMC‐EV, amniotic cell–derived extracellular vesicle. *Source*: Created with Biorender.com.

A schematic representation of the protocol is represented in Figure 1.

### 2.7 Data Analysis

Differences between one versus two treatment cycles were evaluated using the chi‐square test and Fisher's exact test. Statistical significance was set at *p* < 0.05. Statistical analysis was performed using GraphPad InStat 3.00 for Windows (GraphPad Software, La Jolla, CA, USA).

## 3 Results

### 3.1 Characterization of AMC‐EVs (NanoSight, Western Blot Analysis and TEM)

**FIGURE 2:**
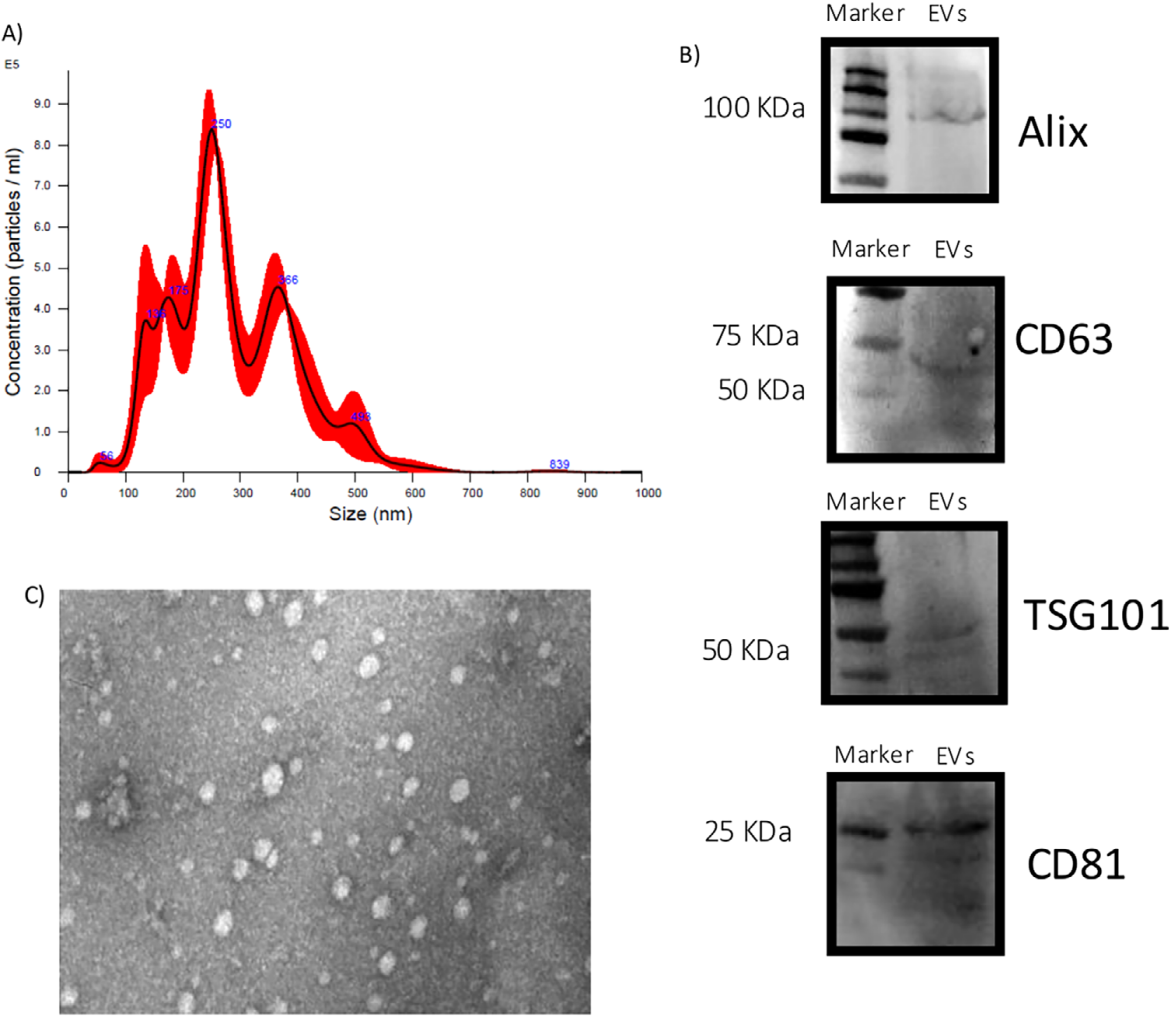
Characterization of AMC‐EVs. (A) Results of Nanosight analysis for AMC‐EV size and concentration; (B) Western blot analysis for Alix and TSG101 as internal markers, and CD63 and CD81 as surface markers expressed by the isolated AMC‐EVs; (C) transmission electron microscopy analysis of AMC‐EVs showing typical morphological characteristics of EVs (scale bar: 0.2 micrometri). Legend: AMC‐EVs, amniotic cell‐derived extracellular vesicles.

NanoSight analysis revealed an average size of 257.7 ± 11.4 nm and a concentration of 1.26 × 10^11^ particles/mL. Given the dimensions obtained, the pool of isolated EVs was composed predominantly of microvesicles (Figure 2A).

After this analysis, samples of 2 × 10^10^ EVs were stored at −80°C until their in vivo use.

Western blot confirmed the presence of Alix and TSG101 internal markers (Figure 2B) and surface markers CD63 and CD81 expressed by the isolated vesicles (Figure 2B).

TEM revealed the spheroid morphology and electron‐dense coat of the EVs (Figure 2C).

### 3.2 Endometrial Biopsy Before AMC‐EV Treatment

The Kenney–Doig classification was used for the histological evaluation of endometrial biopsies. All the mares, except for M6, were affected by chronic endometritis: Seven mares (M1, M2, M4, M8, M9, M11 and M12) were classified IIB category (moderate), and four mares (M3, M5, M7 and M10) were classified IIA category (Table 2). Mare M6 showed a healthy endometrial histological condition despite her history of several failed artificial insemination attempts and classical treatments (anti‐inflammatory drugs and ecbolic agents combined with uterine lavages). For this reason, the veterinarian suggested, including this mare in the study.

**TABLE 2:** Endometrial biopsies before and after amniotic cell–derived extracellular vesicle (AMC‐EV) treatment and pregnancy results.

Mare	Endometrial score before treatment	Endometrial score after treatment	Pregnancy outcome
*Group 1*			
M1	IIB	IIB	Birth of a healthy foal
M2	IIB	IIB	Birth of a healthy foal
M3	IIA	III	Birth of a healthy foal
M4	IIB	IIA	Miscarriage on Month 4
M5	IIA	IIA	Miscarriage on Month 7 due to umbilical cord strangulation
M6	I	I	Not pregnant on the first heat Birth of a healthy foal on the second heat
M7	IIA	IIA	Birth of a healthy foal
*Group 2*			
M8	IIB	IIB	Pregnant; abortion induction by the will of the owner after the first month
M9	IIB	IIB	Birth of a healthy foal
M10	IIA	IIA	Birth of a healthy foal
M11	IIB	IIA	Not pregnant
M12	IIB	IIB	Birth of a healthy foal

### 3.3 Endometrial Biopsy After AMC‐EV Treatment

After the administration of one or two cycles of AMC‐EVs, no adverse reactions or alterations of the reproductive tract occurred, except for mild oedema of the uterine wall.

Generally, the control biopsies at the end of the treatment did not show any improvement in the histological status of the endometrium (Table 2). Only mare M4 (Group 1) and mare M11 (Group 2) switched from Category IIB to IIA. On the contrary, the endometrial condition of mare M3 (Group 1) consistently worsened, going from Category IIA to III, without affecting the positive pregnancy outcome anyway.

### 3.4 Pregnancy Outcome

After artificial insemination with fresh semen, six out of seven mares of Group 1 were pregnant.

All mares of Group 2 were pregnant (Table 2).

There were no statistical differences between one or two AMC‐EV treatment cycles.

Specifically, eight mares (M1–M3, M6, M7, M9, M10, M12) gave birth to healthy foals; Mare M4 encountered a miscarriage at the fourth month of pregnancy; Mare M5's abortion was caused by umbilical cord strangulation of the foal; for Mare M8, abortion was intentionally induced with prostaglandins by the will of the owner after the first month of pregnancy: after several years of failure to conceive, the owner had no expectations of pregnancy on this mare and therefore refused to carry the pregnancy to term, despite having made his mare available for an attempt to conceive.

Interestingly, the only mare that could not establish a pregnancy was M11, which displayed an altered perineal conformation that exposed her to a higher risk of contaminations and uterine infections that might have contributed to the failing outcome.

**FIGURE 3:**
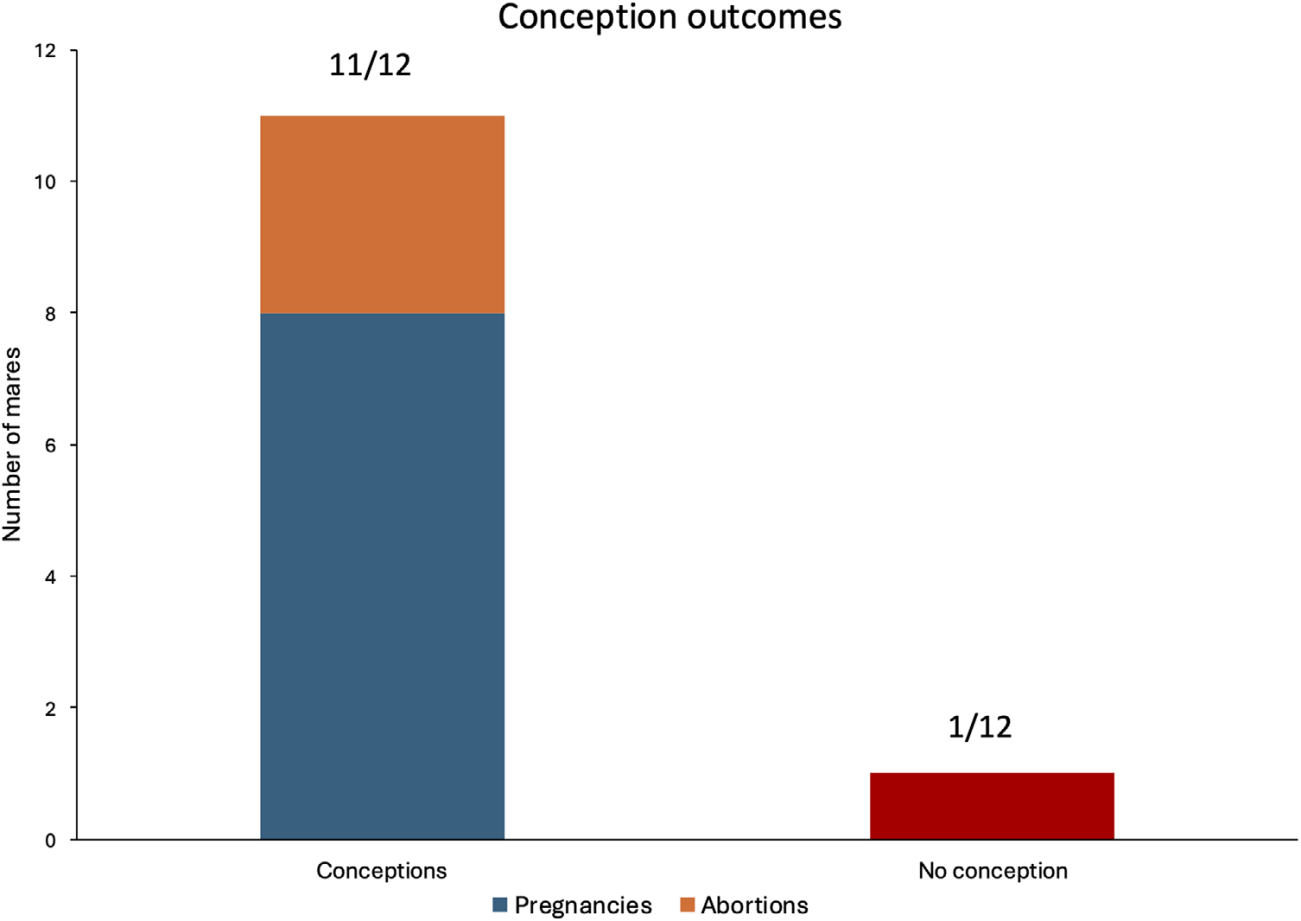
Graphic summary of the conception outcomes.

The pregnancy outcomes are summarized in Figure 3.

## 4 Discussion

Endometrial diseases cause an alteration of the uterine tissue and microenvironment. CDE is characterized by chronic degenerative lesions and fibrosis that severely compromise mares’ fertility. Its aetiopathogenesis is still unclear, but age and repeated pregnancies or acute inflammations might play a significant role in its development. All these factors contribute to an altered endometrial structure that finally results in a deficient interaction between mother and conceptus.

Cellular communication is required in each stage of the reproductive process, from gametogenesis to embryo implantation and development (Mathivanan et al. 2021), and correct foetal–maternal communication is crucial for the establishment of a pregnancy and its outcome. Pre‐implantation is the most delicate phase of gestation, as most embryo losses occur during this stage (Merkt and Günzel 1979; Ginther et al. 1985). During this period, the mutual exchange of signals between maternal and foetal tissues is delicate and crucial for successful adhesion and implantation.

The success of a pregnancy, indeed, requires a molecular dialogue between the embryo and the female reproductive tract, which starts within the oviduct and is maintained until the formation of the placenta. The first known molecules in this paracrine communication were soluble factors, such as cytokines and growth factors: interleukin‐1ß, heparin‐binding epidermal growth factor, integrins and leukaemia inhibitory factor (Bourdiec and Akoum 2014; Bourdiec et al. 2012; Stavreus‐Evers et al. 2005; Jessmon et al. 2009). However, although the soluble secretome is involved in embryo growth, the non‐soluble factors such as EVs are implicated on implantation and embryo development and in the pathophysiology of implantation failure in infertility (Gurung et al. 2020). Trophoblast cells from the interface with the maternal cells and the foetus can secrete EVs to enable the foetus to interact with uterine endothelial cells, whereas the placenta secretes EVs carrying proteins involved in immune modulation, which is essential for the success of pregnancy (Kshirsagar et al. 2012; Mincheva‐Nilsson and Baranov 2014). Indeed, the EVs mediate two‐way trafficking of molecules for embryo–maternal communication. Therefore, the biological function of EVs in regulating early embryo–maternal interaction results from the transfer of critical molecular cargoes to distant or neighbouring recipient cells or tissues.

The altered profile of molecules secreted from embryo or uterus could manifest with the condition of failed conception or failed pregnancy. An increase in EV release by endometrial epithelial cells in this stage of pregnancy has been demonstrated (Tan et al. 2020). EVs contain many molecules, including miRNA (Bruno et al. 2019) and embryo movement is thought to contribute to its recognition through focal adhesion molecule signalling, which is targeted by miRNAs selectively stored into EVs isolated from non‐pregnant mares. This means that this recognition pathway may be repressed in non‐pregnant animals but not in pregnant ones, and it is finely regulated by EV contribution (Klohonatz et al. 2016). Moreover, EVs contained in uterine lumen during the peri‐implantation stage are able to modulate local immune system to mediate immunotolerance to the embryo (Giacomini et al. 2019; Nakamura et al. 2019). Consequently, it is admissible to think that fertility problems might also arise from an incorrect EV production and secretion process that can result in pregnancy failure. Indeed, maternal environmental and physiological factors can directly affect foetal miRNA expression, meaning that dysregulation of miRNA expression may lead to foetal defects or even lifelong consequences (Cai et al. 2017).

miRNAs encapsulated in exogenous EVs could play a significant role in trying to restore intercellular communication, reaching target cells and regulating mRNA and protein expression.

Amniotic mesenchymal EVs have been identified as valuable regenerative medicine tools in previous studies due to their proliferative, anti‐apoptotic, pro‐angiogenic, immunomodulatory and anti‐fibrotic properties (Katsuda and Ochiya 2015). Furthermore, endometrial epithelial cells are able to incorporate AMC‐EVs in vitro, which vehicle into target cells molecules necessary for the secretion of anti‐inflammatory factors (Perrini et al. 2016). A series of miRNAs is selectively compartmentalized into AMC‐EVs and involved in the regulation of the inflammatory mechanism (Lange‐Consiglio et al. 2018).

Given the pivotal contribution of EVs and their effective properties in the modulation of equine uterine environment, this case series tested the efficacy of AMC‐EVs in the restoration of a successful interaction between maternal and foetal tissues during the establishment of pregnancy.

The histopathological examination of endometrial biopsies confirmed that all enrolled mares in this study were affected by CDE, except for M6, which resulted in Category I. The evaluation of the severity of endometritis was performed by the classification of Kenney and Doig (1986), which is a gold standard for endometritis severity, commonly used by private veterinarians to monitor animals of private owners. In the future of experimental studies, endometrial biopsy will certainly give way to other technologies and applications, but Kenney and Doig's contribution will remain a foundation in equine reproduction science.

Eleven mares on a total of 12 successfully established a conception, and 8 mares finally gave birth to healthy foals. The negative outcomes were caused by a miscarriage on the fourth month, a miscarriage on the seventh month due to umbilical cord strangulation and an induced abortion by the will of the owner. Only in the case of mare M11 did the pregnancy assessment result in a negative. Interestingly, this mare also had an altered perineal conformation, which may have caused repeated vulvar contamination that impaired the chances of pregnancy. These results might indicate that the failure of pregnancy in these mares was not due to the inefficacy of the treatment in aiding the foetal–maternal exchange. Indeed, all the other mares carried full‐term pregnancies, despite the several failed attempts prior to the treatment. This suggests that the administration of AMC‐EVs might have led to an improvement of fertility due to the re‐establishment of functional signalling between maternal and foetal tissues. It might be possible to speculate that the integration of AMC‐EVs probably drove a significant restoration of the intercellular communication in an altered uterine environment, where EV production or delivery was defective.

In a previous study, conducted on a single mare affected by CDE, AMC‐EV administration enabled a significant improvement of the histological classification (Lange‐Consiglio et al. 2020). Contrarily, in this case series, none of the enrolled mares showed the same result. Therefore, despite the proven anti‐inflammatory and anti‐fibrotic properties of AMC‐EVs, they were unable to favour the regeneration of damaged endometrial tissue provoked by CDE.

Mare M6 was the only one whose histopathological examination of endometrial biopsy revealed no damage in the endometrial tissue. This mare was enrolled in any case because all the insemination attempts reported in her reproductive history failed. It is possible that her fertility was affected by other unknown factors, but the treatment with AMC‐EVs improved her condition.

There were no significant differences between Groups 1 and 2 treated by single‐ or double‐EV cycle treatment, respectively, in terms of pregnancy outcome. Although pregnancy is a multifactorial outcome, considering the history of the mare enrolled in this study, these results allow us to exclude a potential dose‐dependent effect of this treatment, meaning that a single cycle of EV administration might be sufficient.

Which molecules can be involved in restoring this communication? Although the latest high‐throughput analytical platforms can perform detailed analysis of the lipid, glycolic, proteic and nucleic acid content in EVs (Gézsi et al. 2019), it is still difficult to establish the molecular basis of EV cargo regulating embryo–maternal communication because of the many variables associated with EV isolation and characterization. It is known that embryo EVs transport progesterone‐induced blocking factor 1 that increases IL‐10 production in maternal lymphocytes, sustaining immune responses during pregnancy (Szekeres‐Bartho et al. 2018). In return, placental tissue from the first trimester releases EVs containing proteins with roles in regulating and modulating T‐cell activity to modify the maternal immunological environment.

The administration of EVs likely provided supplementary molecules needed for embryo development and implantation. Moreover, miRNAs can effectively deliver communication between different cell types and tissues under pathophysiological conditions and are actively involved in various cellular activities (Cai et al. 2017). We studied miRNA cargo of AMC‐EVs (Lange‐Consiglio et al. 2018), but no focus was placed on miRNAs involved in paracrine communication mechanisms concerning pregnancy. Currently, we know that AMCs and EVs contain 1285 miRNAs, of which 146 are differently expressed and 17 are more expressed in EVs. Despite the potential regulatory role of these foetal–maternal trafficking miRNAs being widely accepted and their contribution to foetal development and maternal pregnancy‐associated disorders having been proposed, their functional characterization is still lacking (Cai et al. 2017).

Further studies are surely necessary to investigate which are the molecules involved in this process and their mechanism of action.

This study has the limitation of the small number of animals enrolled and the lack of a control group, but this case series includes clinical patients and not experimental or research horses. This is the reason which led to the enrolment of animals with different breeds, age and not control horses. However, considering the large number of past artificial insemination attempts for each animal enrolled in this study, each mare could be considered self‐controlled.

Another limitation of this study is the lack of a dose–response curve to establish a therapeutic dose. To date, in human and veterinary regenerative medicine, there is not a therapeutic dose that depends on the pathology and its severity, the source of MSC, the route, the number or interval of administrations (if there are more administrations) and the use of cells or their secretome.

To choose the amount of EVs for uterine administrations, we referred to a work of Mambelli et al. (2013) that treated mares with endometritis by intrauterine administration of adipose MSCs. In this article, 2 × 10^7^ adipose MSCs were administrated in uterus in 20 mL of physiological solution. As no other indications were present in literature, the amount of EVs secreted by each AMC was calculated using NanoSight data (approximately 1000 EVs from each cell), and the administration dose of AMC‐EVs was set to 2 × 10^10^ to overlap the amount of cells used by Mambelli et al. (2013). Repeated administrations of AMC‐EV were performed because in this study there is a lack of continuous secretion of these molecules by MSCs, and a dose–response effect was evaluated by single or double cycles of administration.

Despite the limitations of this study, the novelty of these preliminary results deserves to be highlighted: For the first time, An intrauterine cell‐free therapy by AMC‐EVs in CDE mares was reported, considering the pivotal role of intercellular communication between the embryo and the endometrium in facilitating a successful pregnancy. EVs influence any stage of reproductive processes, including fertilization (Machtinger et al. 2016). Therefore, the successful outcome of these pregnancies might also have been due to AMC‐EV. At the moment, follow‐up data concerning subsequent pregnancies that could reinforce these results are still not available, as the study was carried out during the 2024 reproductive season.

The encouraging results of this study could suggest that the administration of AMC‐EVs might represent a valid alternative to overcome infertility caused by CDE in mares. A single cycle of two AMC‐EV infusions seems to be sufficient to establish a pregnancy in mares that failed to become pregnant despite several attempts in their past. It is unlikely that these vesicles acted by regenerating the damaged and fibrotic endometrial tissue of these mares. Instead, it would seem possible that they supported and enhanced foetal–maternal communication that was compromised by CDE.

## Author Contributions

Giulia Gaspari performed AMC isolation and EV preparation, characterized AMC‐EVs, evaluated all results and wrote the original draft. Federico Funghi collected allanto‐amniotic membranes, enrolled the animals, performed ultrasonography evaluation, biopsies, EV administration and AI, diagnosed pregnancy and evaluated all results. Carlo Cantile performed biopsy analyses and evaluated all results. Francesco Camillo designed the study, acquired funding, enrolled animals, performed ultrasonography evaluation, biopsies, EV administration, AI and pregnancy diagnosis and evaluated all results. Duccio Panzani enrolled the animals, performed ultrasonography evaluation, biopsies and EV administration and AI, diagnosed pregnancy and evaluated all results. Saverio Maltinti enrolled the animals, performed ultrasonography evaluation, biopsies, EV administration and AI, diagnosed pregnancy and evaluated all results. Diana Fanelli enrolled the animals, performed ultrasonography evaluation, biopsies, EV administration and AI, diagnosed pregnancy and evaluated all results. Rebecca Moroni enrolled the animals, performed ultrasonography evaluation, biopsies, EV administration and AI, diagnosed pregnancy and evaluated all results. Fausto Cremonesi designed the study and acquired funding; all co‐authors provided useful comments on the manuscript. Paola Gagni carried out Nonosigyt, western blot. Anna Lange‐Consiglio designed the study, performed AMC isolation and EV preparation, evaluated all results, carried out statistical analyses and wrote the original draft. All authors have read and approved the final version of the manuscript.

## Funding

This study was funded by PRIN 2022 with the title: *Amniotic mesenchymal extracellular vesicles for equine chronic degenerative endometritis (eCDE) therapy: a model to treat aging and inflammatory uterine pathologies. M.A.R.E.t Mesenchymal Amniotic Regenerative Endometrial therapy* (Project 2022TE87TT_PRIN2022).

## Disclosure

A. Lange Consiglio had full access to all the data in the study and takes responsibility for the integrity of the data and the accuracy of data analysis.

## Ethics Statement

This work involved the use of non‐experimental animals only.

Mares enrolled in this study were made available by private owners after informed consent. The therapeutic protocol proposed was conducted with the approval of the Ethics Committee of the University of Pisa (OPBA_25/2024 of 03/19/2024).

All procedures were conducted following standard veterinary practice and in accordance with the 2010/63 EU directive on animal protection.

## Consent

Signed informed consent was obtained from owners of each animal at study entry.

## Conflicts of Interest

The authors declare no conflicts of interest.

## Data Availability

The data that support the findings of this study are available from the corresponding author upon reasonable request: Open sharing exemption granted by editor for this clinical report.
